# Self-Assembly of NaOL-DDA Mixtures in Aqueous Solution: A Molecular Dynamics Simulation Study

**DOI:** 10.3390/molecules26237117

**Published:** 2021-11-24

**Authors:** Li Wang, Rui Xu, Ruohua Liu, Peng Ge, Wei Sun, Mengjie Tian

**Affiliations:** School of Mineral Processing and Bioengineering, Central South University, Changsha 410083, China; li_wang@csu.edu.cn (L.W.); xrxurui@csu.edu.cn (R.X.); ruohualiu@csu.edu.cn (R.L.); gp-gepeng@csu.edu.cn (P.G.); sunmenghu@csu.edu.cn (W.S.)

**Keywords:** self-assembly behavior, NaOL/DDA mixtures, molecular dynamics’ simulation, aggregation

## Abstract

The self-assembly behaviors of sodium oleate (NaOL), dodecylamine (DDA), and their mixtures in aqueous solution were systematically investigated by large-scale molecular dynamics simulations, respectively. The interaction mechanisms between the surfactants, as well as the surfactants and solvent, were revealed via the radial distribution function (RDF), cluster size, solvent-accessible surface area (SASA), hydrogen bond, and non-bond interaction energy. Results showed that the molecules more easily formed aggregates in mixed systems compared to pure systems, indicating higher surface activity. The SASA values of DDA and NaOL decreased significantly after mixing, indicating a tighter aggregation of the mixed surfactants. The RDF results indicated that DDA and NaOL strongly interacted with each other, especially in the mixed system with a 1:1 molar ratio. Compared to van der Waals interactions, electrostatic interactions between the surfactant molecules were the main contributors to the improved aggregation in the mixed systems. Besides, hydrogen bonds were found between NaOL and DDA in the mixed systems. Therefore, the aggregates in the mixed systems were much more compact in comparison with pure systems, which contributed to the reduction of the repulsive force between same molecules. These findings indicated that the mixed NaOL/DDA surfactants had a great potential in application of mineral flotation.

## 1. Introduction

Mixed surfactants have attracted a great deal of attention from researchers in various fields, including flotation, oil recovery, drug delivery, daily cosmetics, textiles, and pesticide emulsifiers [1,2,3,4,5,6]. Synergistic behavior between two different surfactants results in lower critical micelle concentration (CMC), lower interfacial tension, more stable micelles, and higher solubilization capacity [7,8], which means the combination of surfactants can compensate for each other [9]. The faster formation of micelles in the mixture reduces the dose of surfactants and their damage to the ecological environment. More importantly, detergency, wetting, emulsifying, and effervescent properties of surfactants are also improved [10,11,12,13].

Froth flotation is one of the most effective methods for concentrating valuable minerals from low-grade ores [14,15,16]. In the process, surfactants are used as collectors to enhance the hydrophobicity of valuable minerals. Practices have proven that numerous mixed surfactants show better performance compared to pure surfactants. Among these mixed surfactants, anionic/cationic mixed systems are receiving more and more attention. Many researchers found that the anionic/cationic mixed systems show much higher surface activity compared to other mixed systems due to the strong electrostatic interactions between surfactants [17,18]. Xu et al. [19] proposed that the repulsion between polar head-head was decreased while the hydrophobic bonds between tail-tail were increased when anionic surfactants and cationic surfactants were aggregated. The electrostatic free energy and chemical potential of mixed system were reduced and caused the obvious reduction in CMC. Zhou et al. [20] discovered that anionic/cationic achieved the best result and had the most powerful synergetic effect when they analyzed several mixed systems containing Gemini surfactants.

The mixture of different surfactants enhances the performance of individual surfactants, which primarily depends on the reductive degree of surface tension and the value of CMC. Thermodynamically stable micelles begin to form when the concentration of surfactant is achieved, and the interfacial tension of the solvent reduces to the lowest level. In general, CMC of mixture is found to be much lower than those of a single component, resulting in a higher efficiency of reducing interfacial tension and a lower value of interfacial tension. Furthermore, a lower interfacial tension is beneficial to the production and stabilization of froth in flotation. Especially, the mixed anionic/cationic surfactants show an extremely ideal synergistic effect due to the strong interaction force between molecules. Additionally, the synergistic mechanism has been generally analyzed by numerous parameters’ measurements, such as contact angle, adsorption, zeta potential, Fourier transform infrared spectroscopy (FTIR), viscosity, surface tension, and conductivity [8,18,21,22,23,24,25].

With the eye-catching advances in science and technology, molecular dynamics (MD) has opened up new perspectives for clarifying synergistic theories from the microscopic level [26,27]. Its advantages have been proven and reported by many researchers. For example, an intuitive observation of the three-dimensional structure of the aggregate provides the microscopic behavior and physicochemical properties of the aggregate. In addition, the results and feasibility of the experiment can be predicted and verified, significantly reducing the duration and cost of the experiment. Liu et al. [28] described the micellar structure and adsorption of DDA/DOD (dodecanol) on different quartz surfaces (negatively charged and hydrated) via MD simulations. The mechanism of inhibiting quartz flotation was explained at the molecular level. Wang et al. [29] used MD to explore the microscopic aggregate structure and adsorption behavior of DDA and alcohol with different chain lengths at the water/air surface. The reasons why the properties of mixed surfactants were superior to individual surfactants were clearly elucidated by describing the self-assembling mechanism of different bonded surfactants. The results produced by the simulation were in significant agreement with the results of surface tension. 

Extensive studies have shown that mixed NaOL/DDA surfactants are effectively used for silicate flotation. Flotation separation of silicate ores using NaOL/DDA is adequately mature now and its synergistic mechanism has been reported in a great deal of literature [8,19,30,31]. The flotation process is typically conducted in aqueous solution. As a result, investigating the collective behavior of NaOL/DDA in water is very important for improving flotation performance. Nevertheless, limited research is dedicated to investigating the self-organizing structure and properties of mixed surfactants in water systems. In this paper, the self-aggregation behaviors of NaOL, DDA, and their mixture in aqueous solution were investigated using large-scale molecular dynamics’ simulation. Mixed surfactants of anionic-rich system shows excellent flotation and separation performance on silicate minerals [8,19,30,32,33]. In this regard, the effects of a total molecular number and a range of varied molar ratios on the self-aggregation of mixed NaOL/DDA (NaOL-rich) surfactants in aqueous solution were discussed, respectively.

## 2. Computational Details

### 2.1. Model Construction

**Figure 1:**
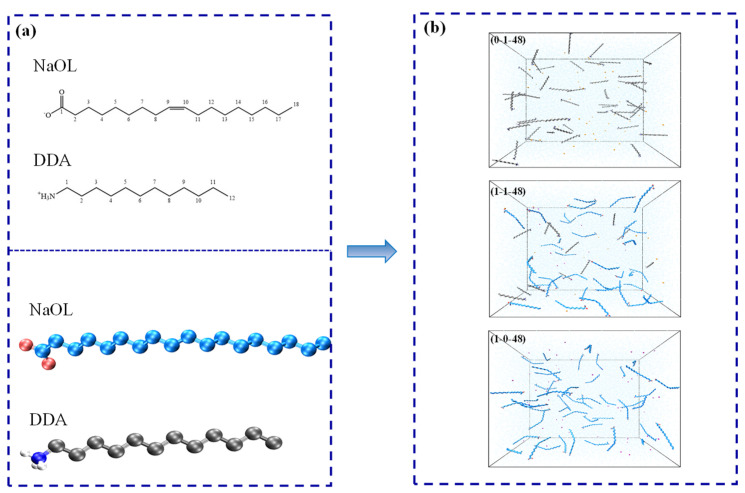
Snapshots of structures. (**a**) Configuration of NaOL and DDA monomers; (**b**) Final configuration of NaOL/DDA (0:1-48, 1:1-48, 1:0-48) systems prepared for simulation.

All simulation processes were performed using the Gromacs 2018 software package. The original coordinate pdb files for anionic surfactant (NaOL) and cationic surfactant (DDA) were generated from the Gaussian09 software package at the B3LYP 6-31G * level [34]. Subsequent MD simulations and analysis of the behavior of surfactants in aqueous solution were completed by virtue of Gromacs2018 and its built-in analytical tools. VMD 1.9.3 was used to visualize the evolution of molecular structures varying with time [29,35,36]. The structure files of NaOL and DDA were imported into ATB (Automated Topology Builder) to establish the initial topology files after optimizing [36,37,38], which was a significant foundation to implement molecular dynamics successfully. Almost all the necessary information to build the molecules was contained in the topology file, including bonded force field parameters (bonds, angles, and dihedrals) and nonbonded force field parameters (atom types and charges). These parameters in the topology file produced by the ATB website were adjusted manually in accordance with the force field of GROMACS 2018 to match the molecular model with force field. The atomic charges of the NaOL and DDA in the topology file were derived from the DFT (density functional theory) calculations. In this paper, the gromos96 53A6 force field was employed to represent the molecular potential parameters of DDA and NaOL [29,35,39], and the molecular structures of NaOL and DDA are shown in Figure 1a. It should be pointed out that the united atom model was used for alkyl chains of surfactants, including CH_3_ and CH_2_ groups. The flexible simple point charge (SPC), which belonged to a common, equilibrated, three-point water model, was utilized to describe the interactions between water molecules [40].

First, we created simulation cubic boxes with sizes of 9.8, 6.3, and 11.1 nm in three directions. Periodic boundary conditions (PBC) were adopted in the x, y, and z dimensions to make the simulation system realistic. A series of models were obtained via combining the different numbers of NaOL and DDA randomly including the mole ratios of 0:1, 1:1, 2:1, 3:1, 4:1, 5:1, 6:1, 7:1, and 1:0, with the four total molecular numbers of 48, 96, 144, and 196. Afterwards, the simulation boxes were filled with water. In the end, a certain number of sodium ions and chlorine ions was added to these boxes to replace water molecules as counterions All the molecules were randomly inserted into the box at the beginning of the simulation. Figure 1b displays the snapshots of the final boxes before starting the molecular dynamic simulation. The numbers of NaOL, DDA, counterions, and water molecules in various systems are also demonstrated (Table 1). Ultimately, the systems with a total molecular number of 48 were used to explore the self-assembly behavior of two surfactants with different molar ratios, and the system with a molar ratio of 1:1 was used to analyze the influence of the total molecular numbers on aggregates (48, 96, 144, and 192 molecules). 

### 2.2. Equilibrium Process

#### 2.2.1. Energy Minimization

**Figure 2:**
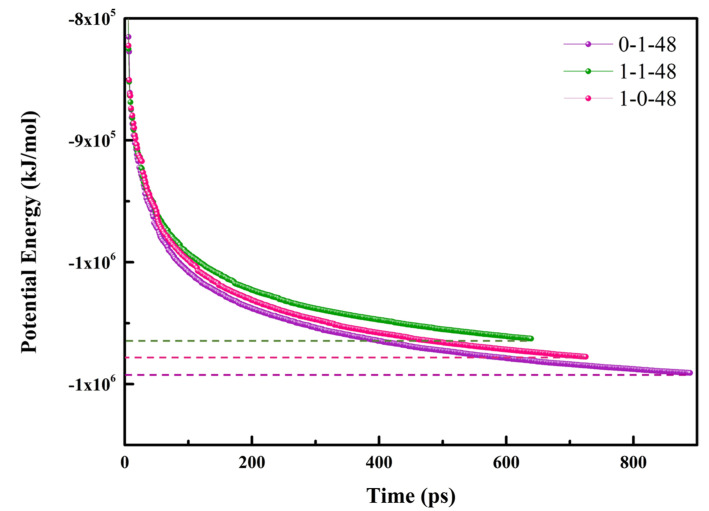
Potential energy of NaOL/DDA in energy minimization process.

Energy minimization is a prerequisite for successful molecular simulations. All molecules were randomly added to the box, which may cause anomalous contact between atoms and improper shape. These defects of initial structures could be eliminated by energy minimization to obtain optimized geometry. In our work, the steepest descent algorithm was applied during the energy minimization process. The maximum force (F_max_) was set to 1000 in order to assess whether energy minimization was successful. When F_max_ of a system was less than 1000 kJ mol^−1^nm^−1^, the process would be terminated, as shown in Figure 2. The maximum position movement of energy optimization was set to 0.01 ns and the whole process could perform up to 50,000 steps, which was adequate for all simulation systems. After energy minimization, the stable tendency for potential energy of all systems indicated the energy of systems was minimum. 

#### 2.2.2. Equilibration

The system first achieved a certain temperature with the NVT canonical ensemble. The temperature was desired to stabilize at 300 K through NVT ensemble. In this paper, we used the Leapfrog algorithm with a time step of 2 fs to integrate the equations of motion. All bond lengths were constrained by the system’s LINCS algorithm. The cutoff radius of both electrostatic interactions and the van der Waals (vdW) interactions was 1.0. The PME (Particle Mesh Ewald) was applied to calculate the long-range electrostatics. The NVT ensemble was performed at 300 K in 1,000,000 steps [27,41]. After every 5000 steps, the information, including coordinates, velocities, and energies, was exported. The Verlet algorithm with the type of grid was used for buffered neighbor searching during the simulation process. The Velocity-rescale thermostat with the time constant of 0.1 ps was adopted to maintain the temperature. Periodic boundary conditions were adopted in all three directions, x, y, and z. The initial velocity of the simulation used a random number generated by Maxwell.

A simulation system, which was closer to the realistic system, could be created by controlling the temperature and pressure. Followed by the balance of temperature, the pressure was stabilized by NPT equilibration. In our study, the pressure of the systems was maintained at 1 bar using the Berendsen thermostat with the time constant of 3.0 ps [27,41]. The output information was saved every 50,000 steps. Similarly, LINCS algorithm was used to constrain all bonds; the setting of vdW interactions and long-range electrostatic interactions remained the same with NVT ensemble. The periodic boundary conditions were also utilized in x, y, and z orientations. The whole equilibrium process was continued for 20 ns. 

## 3. Results and Discussion

### 3.1. Aggregation Behavior

**Figure 3:**
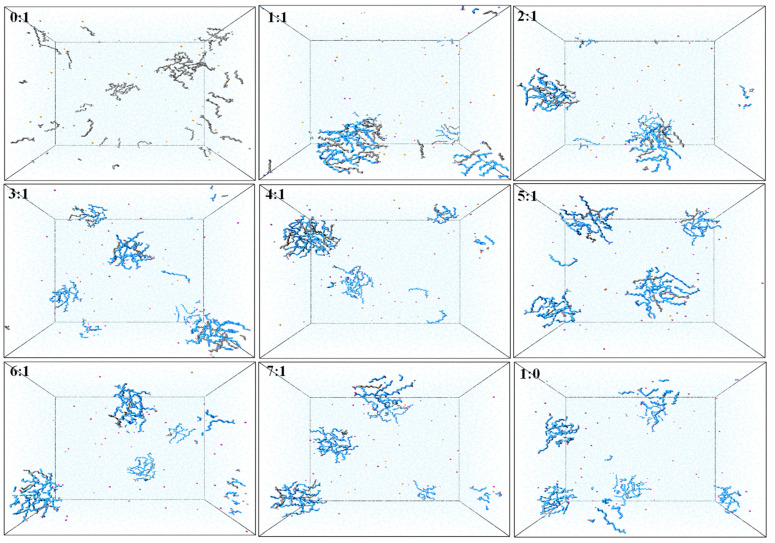
Snapshots of aggregates formed by NaOL and DDA molecules in systems with different NaOL/DDA molar ratios (0:1, 1:1, 2:1, 3:1, 4:1, 5:1, 6:1, 7:1, and 1:0) at the final state of simulation.

Snapshots of molecular configuration in the simulation of the final state (Figure 3) were conducted to explore the morphology of different NaOL/DDA molar ratios in aqueous solution. The total molecular number of NaOL and DDA was 48 in each simulation system. We were able to intuitively observe the degree of aggregation varying with different NaOL/DDA molar ratios from these snapshots. In these nine systems, DDA hardly formed aggregates and had the largest number of monomers, which were disorderly distributed in the aqueous solution. Although the aggregates formed were looser, individual NaOL molecules were more likely to self-associate to form the spherical aggregates than DDA molecules. In the mixed systems of NaOL and DDA, different numbers of NaOL and DDA molecules appeared alternately in an aggregate, and there were a few single NaOL complexes or DDA complexes. The two types of molecules generated the intermolecular interactions and decreased the critical micelle concentration (CMC), promoting the extent of molecule aggregation. The better tendency of mixed aggregation was consistent with the experimental results studied by Xu et al. [19]. Similarly, a small number of NaOL or DDA monomers also existed in the solution. Among simulation systems that formed polymers, the hydrophobic carbon tails contacted each other, which composed the interior structure of the association. While the head groups of NaOL and DDA distributed on the surface of spherical aggregate, all the aggregates existed stably in the aqueous solution. The above phenomenon indicated that the interaction force between head groups and the hydrophobic interaction between alkyl tails was the essential driving force for self-assembling. Moreover, several small aggregates were observed simultaneously in most systems. The optimal extent of NaOL/DDA aggregation was obtained and a larger aggregate was easier to generate in the mixed system with the molar ratio of 1:1. 

**Figure 4:**
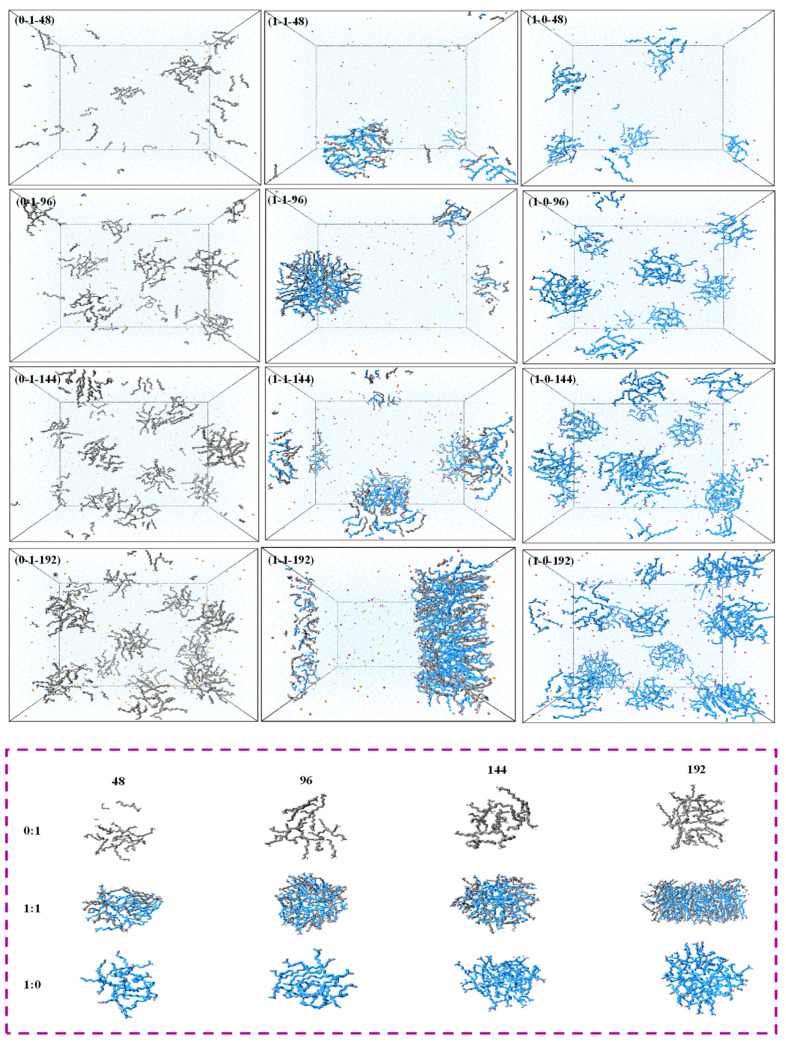
Snapshots of NaOL/DDA molecules in systems with different total numbers of molecules.

To further discuss the effect of the total number of molecules on aggregation, we derived snapshots of a different total number of molecules (48, 96, 144, and 192) in a pure system and a 1:1 mixed system, as presented in Figure 4. The aggregation of surfactants increased with the increasing total molecular numbers. However, instead of forming particularly large aggregates, some small aggregates were dispersed in the system. The mixed system with the molar ratio of 1:1 showed a visible difference compared to the pure system. Most molecules formed large and dense aggregates with the molecular number of 96. When the total number added to 144, the monomers formed prominent aggregates and a few free monomers were found in the solution. An interesting problem was that the aggregates further recombined and transitioned from spherical micelles to rod micelles with the total molecular number up to 192. This indicated that the current concentration had reached 10 times that of CMC [42]. The results clearly demonstrated that the 1:1 molar ratio system had the lowest CMC value. In summary, the increase in the total number of molecules favored the formation of larger and tighter clusters. 

**Figure 5:**
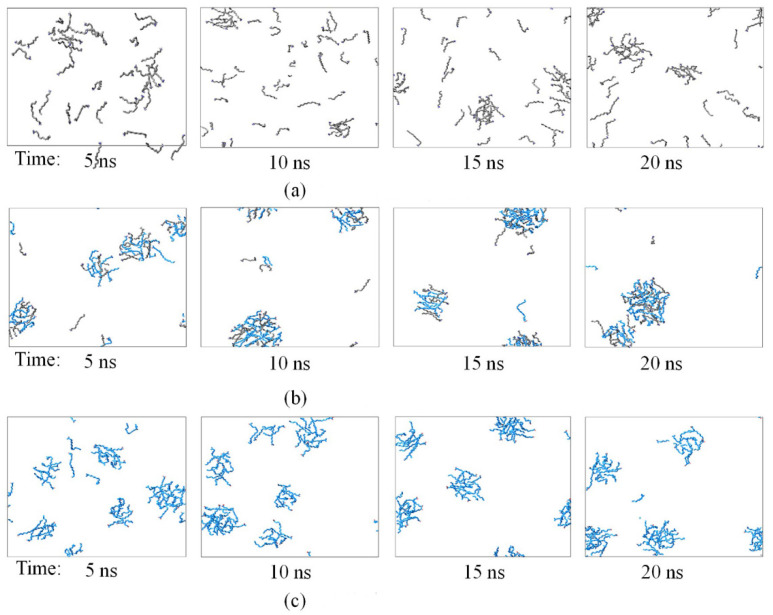
Snapshots of NaOL/DDA systems at different simulation times for (**a**) NaOL/DDA_0:1-48, (**b**) NaOL/DDA_1:1-48, and (**c**) NaOL/DDA_1:0-48.

To further explore the aggregate state of molecules, snapshots of clusters both in pure systems and 1:1 system, with a molecular number of 48, were investigated (Figure 5). We found that the increasing simulation time led to a higher level of aggregation in both pure systems and mixed system. The aggregation rate of the pure NaOL system and the mixed system was much faster compared with pure DDA system, of which the obvious clusters appeared at 5 ns, especially in the 1:1 mixed system. It indicated that the aggregate intensity between different surfactant molecules was larger than the individual surfactant molecule. The quick aggregation of pure NaOL may be related to the longer hydrophobic tail chains, and the strong intermolecular interactions contributed the association between DDA and NaOL.

### 3.2. Radial Distribution Function

**Figure 6:**
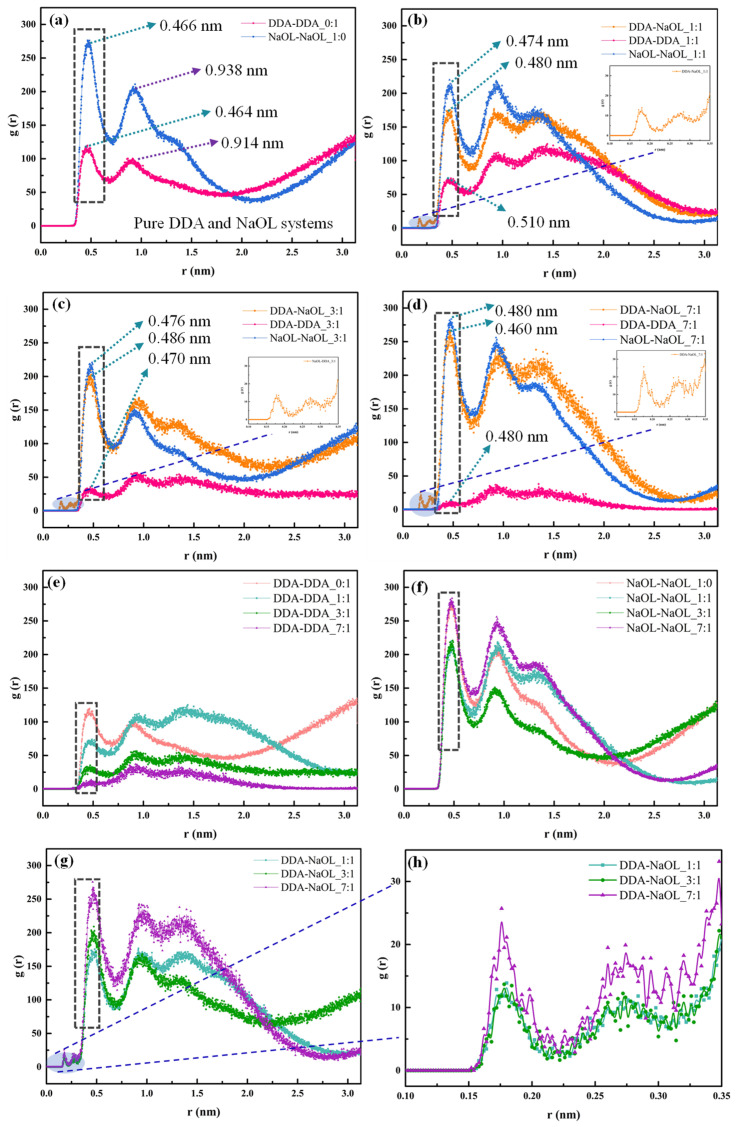
Radial Distribution Function of NaOL and DDA monomers averaged over the last 2 ns of MD simulations for (**a**) NaOL/DDA_0:1 and NaOL/DDA_1:0 systems, (**b**) NaOL/DDA_1:1 system, (**c**) NaOL/DDA_3:1 system, and (**d**) NaOL/DDA_7:1 system. (**e**) are RDFs between DDA monomers in NaOL/DDA_0:1 system, NaOL/DDA_1:1 system, NaOL/DDA_3:1 system and NaOL/DDA_7:1 system. (**f**) are RDFs between NaOL monomers in NaOL/DDA_1:0 system, NaOL/DDA_1:1 system, NaOL/DDA_3:1 system and NaOL/DDA_7:1 system. (**g**) are RDFs between DDA and NaOL monomers in NaOL/DDA_1:1 system, NaOL/DDA_3:1 system and NaOL/DDA_7:1 system. (**h**) are RDFs between DDA and NaOL monomers within 0.35 nm in NaOL/DDA_1:1 system, NaOL/DDA_3:1 system and NaOL/DDA_7:1 system.

The value of radial distribution functions (RDF) calculated in this work was used to represent the possibility that the concerned group appeared in a shell dr at the distance r from the surface of the reference group. Figure 6a shows the RDF of DDA-DDA and NaOL-NaOL in pure DDA and NaOL system. The first peak appeared in both DDA and NaOL systems at around 0.46 nm, which was the most obvious peak. The presence of the peak demonstrated the highly molecular aggregation of DDA or NaOL monomers in pure systems. Compared with the pure NaOL system, the intensity of the peak significantly decreased in the pure DDA system, indicating that the molecular aggregation tendency of the DDA monomer was much lower than that of the NaOL monomer. Figure 6b–d shows the intensity of the interaction between the same molecular model and disparate molecular models in a series of mixed systems with various molar ratios. For the three mixed systems, the magnitude of the first peak followed the order of NaOL-NaOL ≈ DDA-NaOL > DDA-DDA. This finding suggested that the aggregation between NaOL molecules was strongest when DDA and NaOL molecules existed in the solvent simultaneously. However, the coalescence between two different molecules was only slightly lower than that of the NaOL molecules, and the DDA molecules had the weakest tendency of aggregation. What is attractive is that the second and the third peaks’ heights of DDA-NaOL were higher than NaOL-NaOL in the mixed systems with the molar ratio of 3:1 and 7:1, respectively. This illustrated that the aggregate tendency between DDA and NaOL was stronger than NaOL-NaOL. Additionally, the progressive reduction in the first peak’s height from system NaOL/DDA_7:1 to systems NaOL/DDA_3:1 and NaOL/DDA_1:1 indicated the molecular number of NaOL had influence on the aggregation degree of DDA and NaOL(Figure 6g). This is interesting to note that DDA and NaOL had the trend to aggregate at around 0.18 nm. The aggregation tendency between the same molecules in different systems is given in Figure 6e,f. Moreover, the optimal peak of DDA-DDA was obtained with the molar ratio of 1:1. It was because the head groups of DDA and NaOL had opposite charges, which reduced the repulsion force between the head groups of the same molecule. The degree of aggregation between DDA molecules was correspondingly the most excellent with the molar ratio of 1:1. 

**Figure 7:**
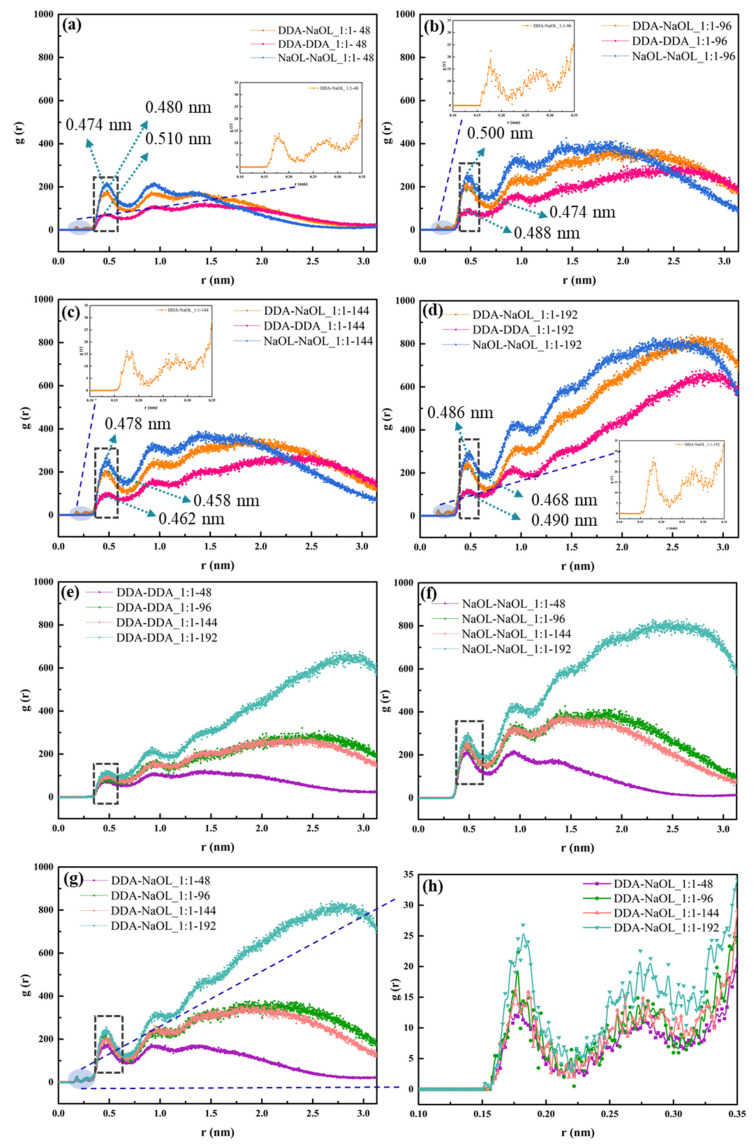
Radial Distribution Function of DDA and NaOL monomers averaged over the last 2 ns of MD simulations for (**a**) NaOL/DDA_1:1-48 system, (**b**) NaOL/DDA_1:1-96 system, (**c**) NaOL/DDA_1:1-144 system, and (**d**) NaOL/DDA_1:1-192 system. (**e**) are RDFs between DDA monomers in NaOL/DDA_1:1-48 system, NaOL/DDA_1:1-96 system, NaOL/DDA_1:1-144 system and NaOL/DDA_1:1-192 system. (**f**) are RDFs between NaOL monomers in NaOL/DDA_1:1-48 system, NaOL/DDA_1:1-96 system, NaOL/DDA_1:1-144 system, and NaOL/DDA_1:1-192 system. (**g**) are RDFs between DDA and NaOL monomers in NaOL/DDA_1:1-48 system, NaOL/DDA_1:1-96 system, NaOL/DDA_1:1-144, and NaOL/DDA_1:1-192 system. (**h**) are RDFs between DDA and NaOL monomers within 0.35 nm in NaOL/DDA_1:1-48 system, NaOL/DDA_1:1-96 system, NaOL/DDA_1:1-144 system, and NaOL/DDA_1:1-192 system.

The aggregation mechanism of a 1:1 mixed system was investigated due to its interesting characteristics. Figure 7 shows the radial distribution function of DDA and NaOL in systems with four total molecular numbers (48, 96, 144, and 192). The magnitude of the first peak between surfactants in different systems still followed the order of NaOL-NaOL ≈ DDA-NaOL > DDA-DDA. This result indicated a higher tendency of aggregation between NaOL monomers and DDA-NaOL. When the total molecular number reached 192, the increasing value of the radial distribution function demonstrated the transition from spherical to rod-like micelles. Additionally, the molecular aggregation of the first layer, including the same surfactant and the different surfactants, had a negligible relationship with the augment of the total molecular number. Nevertheless, the magnitude of aggregates was exaggerated with the increasing total molecular number.

### 3.3. The Size Distribution of Cluster

**Figure 8:**
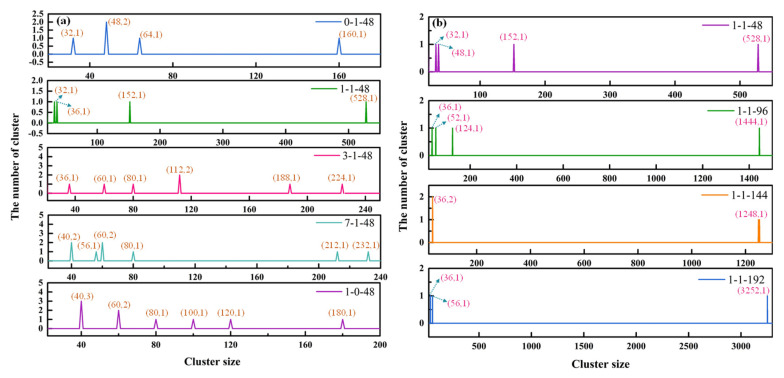
Cluster size containing more than 32 atoms over the last frame of MD simulations for systems: (**a**) Cluster size for NaOL/DDA_0:1-48, NaOL/DDA_1:1-48, NaOL/DDA _3:1-144, NaOL/DDA_7:1-48 and NaOL/DDA_1:0-48; (**b**) Cluster size for NaOL/DDA_1:1-48, NaOL/DDA_1:1-96, NaOL/DDA_1:1-144 and NaOL/DDA _1:1-196.

To further determine the effect of molar ratio on the size distribution of aggregates, the number of clusters in the last frame of the systems was calculated, as shown in Figure 8a. It can be calculated that the number of 16-atom clusters in a pure DDA system reached 26, and the number of 20-atom clusters in a pure NaOL system was about 12. These results indicate the presence of large amounts of free monomers in the water and that NaOL had a superior tendency of aggregation compared with DDA. Basically, some oligomers (≤10 monomers) were observed in the solution. The relative abundance of free monomers was obviously decreased in the mixed systems. Compared with pure systems, the mix of DDA and NaOL contributed to the aggregation of molecules and the formation of larger clusters. In addition, the number of atoms could reach 528 at 1:1 molar ratio. This finding proved that the highest level of aggregation appeared when the molar ratio was 1:1.

Figure 8b shows the number of clusters in the last frame of a 1:1 system with a total of four molecular numbers. The augment of the total number of molecules reduced the number of free monomers and promoted the formation of larger aggregates. In addition to the enlargement of the individual aggregates, several relatively small micelles approached each other and recombined into larger flocs. In consequence, the aggregates became bigger with the aid of the total molecular numbers.

### 3.4. Solvent-Accessible Surface Area

**Figure 9:**
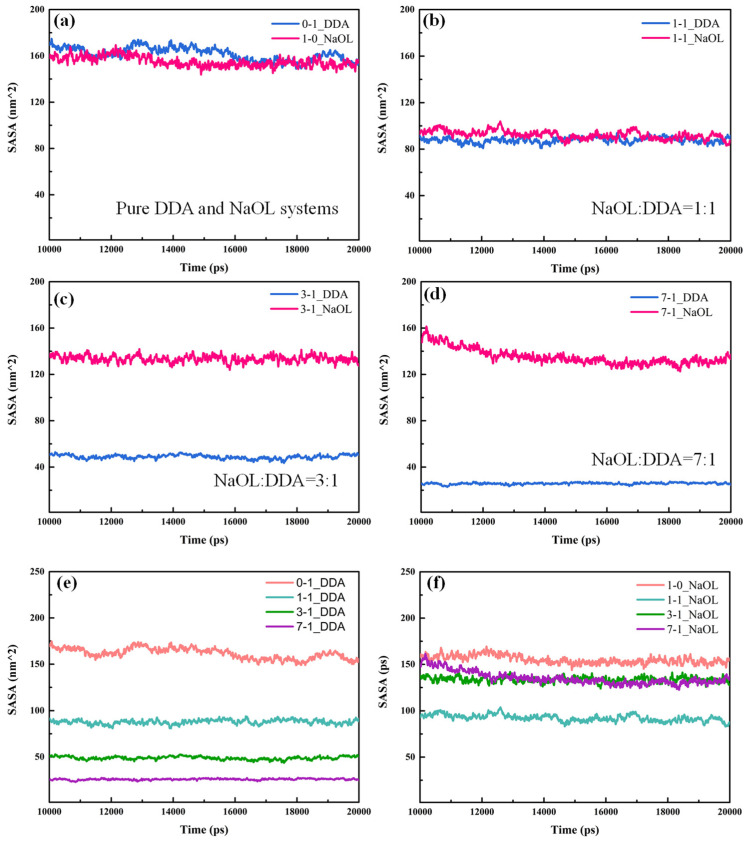
Solvent-accessible surface area (SASA) of DDA and NaOL molecules calculated over the last 10 ns for (**a**) NaOL/DDA_0:1 and NaOL/DDA_1:0 systems, (**b**) NaOL/DDA_1:1 system, (**c**) NaOL/DDA_3:1 system, and (**d**) NaOL/DDA_7:1 system. (**e**) are SASA between of DDA molecules in NaOL/DDA_0:1 system, NaOL/DDA_1:1 system, NaOL/DDA_3:1 system and NaOL/DDA_7:1 system. (**f**) are SASA of NaOL molecules in NaOL/DDA_1:0 system, NaOL/DDA_1:1 system, NaOL/DDA_3:1 system and NaOL/DDA_7:1 system.

Solvation of DDA and NaOL molecules in aqueous solution had a significant effect on the aggregation of molecules in the solution. The solvent-accessible surface area (SASA) [39], which reflected the area of DDA or NaOL molecules contacting with solvation, was analyzed to investigate the diversity on aggregation of DDA and NaOL in our systems (Figure 9). The SASA of these systems remained stable in the end, which meant the self-organization was stabilized. In the pure system, the SASA value of NaOL was slightly lower than DDA (Figure 9a), which indicated NaOL monomers were easier to aggregate and the structure of NaOL aggregate was tighter; thus, water molecules had difficulty entering the interior of aggregates. As the molar ratio of the mixed system was 1:1, the solvation of DDA and NaOL was almost similar. Quite differently, the SASA value of NaOL was higher than that of DDA in other mixed systems (Figure 9c,d), including the molar ratios of 3:1 and 7:1; the SASA difference between two types of molecules was expressly pronounced. The results showed that, as the number of NaOL monomers increased in systems with various molar ratios, one DDA monomer was surrounded by more NaOL molecules. Thus, the number of water molecules around DDA was reduced because of the strong interaction force between NaOL and DDA molecules, which caused a decreased degree of solvation for DDA. While more NaOL monomers participated in the formation of aggregates, more hydrophilic head groups of NaOL distributed on the surface of mixed aggregates, causing the higher solvation of NaOL, as shown in Figure 9e,f. Moreover, the SASA value of DDA and NaOL in mixed systems was lower than that in pure systems, which was related to the tighter aggregation of monomers in the mixed system. The above results proved that DDA and NaOL monomers intersected with each other and formed aggregates in mixed systems. A large electrostatic interaction was generated between DDA and NaOL molecules due to opposite charge of the polar head groups for two surfactants, which led to more compact polymer structures. Thereby, DDA and NaOL became more hydrophobic in mixed systems compared with pure DDA or NaOL system. Nevertheless, the lowest SASA value in a system with the molar ratio of 1:1 for NaOL indicated that aggregates in this mixed system were optimal and the interaction force between DDA and NaOL was strongest. This result was consistent with RDF peaks for systems, as observed in Figure 6.

**Figure 10:**
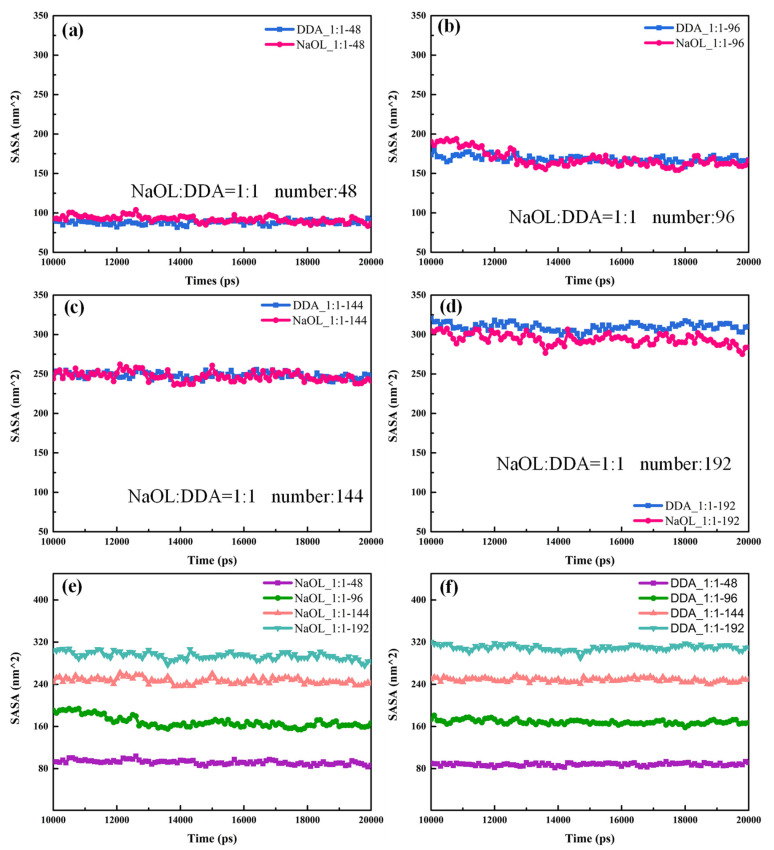
Solvent-accessible surface area (SASA) of DDA and NaOL molecules calculated over the last 10 ns for (**a**) NaOL/DDA_1:1-48 system, (**b**) NaOL/DDA_1:1-96 system, (**c**) NaOL/DDA_1:1-144 system, and (**d**) NaOL/DDA_1:1-192 system. (**e**) are SASA of NaOL molecules in NaOL/DDA_1:1-48 system, NaOL/DDA_1:1-96 system, NaOL/DDA_1:1-144 system and NaOL/DDA_1:1-192 system. (**f**) are SASA of DDA molecules in NaOL/DDA_1:1-48 system, NaOL/DDA_1:1-96 system, NaOL/DDA_1:1-144 system and NaOL/DDA_1:1-192 system.

The SASA values for systems with different numbers of molecules are shown in Figure 10. As the total number of molecules increased, the distinction of the solvation between DDA and NaOL in the same system was not obvious. In addition, the SASA value of DDA and NaOL in different systems followed the order of DDA_1:1-192 > DDA_1:1-144 > DDA_1:1-96 > DDA_1:1-48 and NaOL_1:1-192 > NaOL_1:1-144 > NaOL_1:1-96 > NaOL_1:1-48, respectively, indicating that the larger aggregates may have formed with the increase of total molecular number; hence, the hydrophilic head groups that distributed on the surfaces of aggregates were added, which led to a more solvation of surfactant molecules.

### 3.5. Hydrogen Bond

The hydrogen bond played an important role in the process of molecular aggregation. The average number of hydrogen bonds between the molecular model, as well as the molecular model and water, was calculated to determine the influence of DDA and NaOL molecules on the stability of aggregate with different molar ratios (Table 2). In our simulation, r_DA_ (distance between donor-acceptor) ≤ 0.35 nm and θ_ADH_ (angle of acceptor-donor-hydrogen) ≤ 30° were chosen to use as the standard for calculating a hydrogen bond [36,43]. We observed that hydrogen bond did not form between identical molecules in all systems, such as DDA-DDA and NaOL-NaOL. It was attributed to the absence of a lone electron pair in the polar head groups of DDA and the lack of donor between NaOL molecules. The results showed that the water solubility of NaOL was higher than that of DDA, which was likely due to the further quantity of lone electron pairs in the group of COO^−^. In a mixed system, hydrogen bonds between DDA an NaOL were found, for which a few tiny peaks, less than 0.35 nm, appeared and located at almost the same positions in Figure 6g. Additionally, as the number of NaOL increased, the hydrogen bonds formed by DDA and NaOL molecules decreased from 11.06 to 4.32. These results reflected that the aggregates formed in a 1:1 molar ratio had the tightest structure. In addition, the mixture of the two surfactants reduced the hydrogen bonds between the single surfactant and the solvent compared to the pure system. The number of hydrogen bonds in the DDA-solvent decreased from 128.52 to 12.53, and the number of hydrogen bonds in NaOL-solvent decreased from 294.39 to 129.26. Therefore, when DDA and NaOL molecules presented in the system at the same time, the solvation of two surfactants reduced and the degree of binding for DDA and NaOL molecules improved. These were consistent with our observations from RDFs. 

Table 3 shows the average number of hydrogen bonds among DDA and NaOL molecules, as well as surfactant and water molecules in a 1:1 mixing system. As the total number of molecules increased from 48 to 192, the DDA-solvent hydrogen bonds increased from 55.93 to 190.81, and the NaOL-solvent hydrogen bond increased from 129.02 to 436.71. At the same time, the number of hydrogen bonds between DDA and NaOL increased from 11.06 to 80.86 when 192 surfactant molecules were added to the system. These results explain the increase in SASA values, as shown in Figure 10e,f, and further indicate that the increase in total number of molecules promoted the aggregation of surfactant molecules. 

### 3.6. Nonbonding Interaction Energies

**Figure 11:**
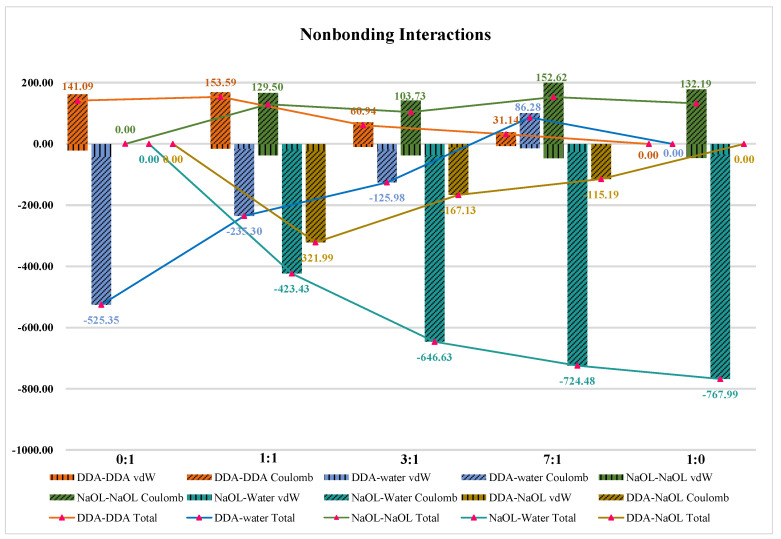
**The** vdW and Electrostatic Interaction Energies between DDA-DDA, DDA-water, NaOL-NaOL, NaOL-water, DDA-NaOL averaged over the last 2 ns of MD simulations for NaOL/DDA_0:1-48 system, NaOL/DDA_1:1-48 system, NaOL/DDA_3:1-48 system, NaOL/DDA_7:1-48 system and NaOL/DDA_1:0-48 system.

The presence of hydrogen bonds indicated that the energy of the nonbonding interaction between the surfactant and the solvent and between surfactants had a significant effect on the self-assembly of the surfactant. Interactions between surfactant molecules and solvents led to solvation of surfactants, while interactions between surfactant molecules were conductive in the formation of aggregates. Figure 11 summarizes the nonbonding interaction energies between surfactants and solvent as well as surfactants, including vdW and electrostatic interaction energies. The results showed that the electrostatic interaction energies were dominant in all systems. The nonbonding interactions between surfactant molecules and solvent followed the order of NaOL-water > DDA-water in all systems, which was related to the more hydrogen bonds formed by NaOL and water. Moreover, the interactions between the same surfactant molecules were repulsive due to the overlapping electrical properties of the polar head groups. Examining the interactions in mixed systems, we clearly found that the nonbonding interaction energies between DDA and NaOL were particularly strong due to the opposite charge of headgroups for two surfactants, especially when the number of DDA and NaOL was equal in mixed system. The nonbonding interaction energies achieved 321.99 kJ/mol, which was larger than that of other mixed systems. In addition, the mix of DDA and NaOL decreased the interactions of surfactants and water. The interaction of NaOL-water reduced from 767.99 kJ/mol to 423.42 kJ/mol, while the DDA-water interaction decreased from 525.35 kJ/mol to 86.28 kJ/mol Thus, the degree of solvation for NaOL and DDA was reduced. Meanwhile, the repulsion between the same molecules, such as DDA-DDA and NaOL-NaOL, weakened from 141.09 kJ/mol and 132.19 kJ/mol to 31.14 kJ/mol and 129.50 kJ/mol, respectively, which improved the compactness of the mixed aggregates. Overall, all results further demonstrated that two different surfactants were easy to interpenetrate to form aggregates in mixing systems. This was due to the large attractive force between the two surfactants with opposite charges of the head group and some water molecules being removed from the agglomerates, resulting in less contact between water molecules and surfactants. The closer aggregate structures were formed. Especially in the system with a molar ratio of 1:1, the aggregate formed was the most compact.

**Figure 12:**
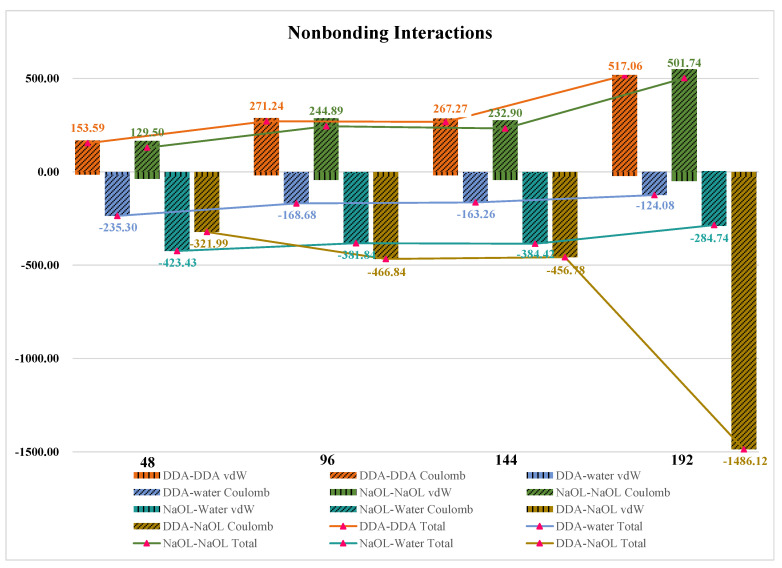
**The** vdW and Electrostatic Interaction Energies between DDA-DDA, DDA-water, NaOL-NaOL, NaOL-water, DDA-NaOL averaged over the last 2 ns of MD simulations forNaOL/DDA_1:1-48 system, NaOL/DDA_1:1-96 system, NaOL/DDA_1:1-144 system and NaOL/DDA_1:0-192 system.

It is seen from Figure 12 that the nonbonding interactions between surfactant-water followed the order of surfactant-water_48 > surfactant-water_96 ≈ surfactant-water_144 > surfactant-water_192. The nonbonding interactions between DDA and NaOL increased in the order of DDA-NaOL_48 < DDA-NaOL_96 ≈ DDA-NaOL_144 < DDA-NaOL_192, consistent with the results of Table 3. Consequently, the results in Figure 12 confirmed that the aggregate tendency of surfactants became larger and the association between DDA and NaOL enhanced with the increase of total molecular number. 

## 4. Conclusions

In summary, the results of a systematic study conducted to investigate the self-assembling behavior of mixed anionic-cationic surfactants in aqueous solution was reported. DDA and NaOL molecules form aggregates in solution and, compared to pure DDA or NaOL systems, the two surfactant monomers interpenetrate and aggregate to form mixed aggregates. This is due to the strong nonbonding interaction forces between the head groups with opposite charges of DDA and NaOL and the increase of hydrophobic tail-tail binding. We also found that the higher the total number of systems (pure DDA systems, pure NaOL systems, and NaOL/DDA mixed systems), the higher the aggregation level, the smaller the number of free monomers, and the more compact the structure of the formed aggregates. What is more, the increase in total number was beneficial for the formation of larger aggregates.

## Figures and Tables

**Table 1:** Details in each simulation system.

NaOL/DDA	Total Number	OL^−^	DDAH	Na^+^	CL^−^	H_2_O
0:1	48	0	48	0	48	21824
	96	0	96	0	96	21335
	144	0	144	0	144	20800
	192	0	192	0	192	20317
1:1	48	24	24	24	24	21754
	96	48	48	48	48	21166
	144	72	72	72	72	20574
	192	96	96	96	96	20007
2:1	48	32	16	32	16	21713
3:1	48	36	12	36	12	21717
4:1	50	40	10	40	10	21677
5:1	48	40	8	40	8	21674
6:1	49	42	7	42	7	21688
7:1	48	42	6	42	6	21712
1:0	48	48	0	48	0	21660
	96	96	0	96	0	21034
	144	144	0	144	0	20346
	192	192	0	192	0	19716

**Table 2:** Number of hydrogen bonds between molecules averaged over the last 2 ns of simulation for systems with different molar ratios.

System	DDA-Solvent	NaOL-Solvent	DDA-NaOL
NaOL/DDA			
0:1	128.52	0.00	0.00
1:1	55.93	129.02	11.06
3:1	28.12	214.10	5.44
7:1	12.53	250.06	4.32
1:0	0.00	294.39	0.00

**Table 3:** Number of hydrogen bonds between molecules averaged over the last 2 ns of simulation for systems with different total molecular numbers.

Number	DDA-Solvent	NaOL-Solvent	DDA-NaOL
48	55.93	129.02	11.06
96	107.52	246.62	27.91
144	159.48	370.57	40.14
192	190.81	436.71	80.86

## Data Availability

Not applicable.
